# Dual ureteral stent placement after redo laser endoureterotomy to manage persistent ureteral stricture

**DOI:** 10.1002/iju5.12152

**Published:** 2020-03-06

**Authors:** Masahiko Isogai, Shuzo Hamamoto, Kenichi Hasebe, Keitaro Iida, Kazumi Taguchi, Ryosuke Ando, Atsushi Okada, Takahiro Yasui

**Affiliations:** ^1^ Department of Nephro‐urology Nagoya City University Graduate School of Medical Sciences Nagoya Japan

**Keywords:** balloon dilation, dual stents placement, laser endoureterotomy, ureteral stricture, ureteroscopic lithotripsy

## Abstract

**Introduction:**

Endourological intervention is a minimally invasive approach for the management of ureteral strictures. Contraindications to this approach include active infection, strictures of sizes >2 cm, and failure of endoureterotomy. This report demonstrates a case of successful dual stent placement after redo endoureterotomy.

**Case presentation:**

A recurring ureteral stricture in a 69‐year‐old woman, who had undergone ureteroscopic lithotripsy for a right ureteral calculus 60 months earlier, was successfully managed by redo endoureterotomy. The procedure involved insertion of dual ureteral stents after endoluminal incision and balloon dilation. Ureteral stents were removed 8 weeks after the operation. No significant complications or signs of stricture were observed 42 months after endoscopic repair.

**Conclusion:**

This minimally invasive and effective technique of dual ureteral stent placement following laser endoureterotomy successfully managed the recalcitrant ureteral stricture in a case with failed single stent placement following endoureterotomy.

**Abbreviations & Acronyms**
CT: computed tomography
DJ: double J
DTPA: dynamic ^99m^Tc‐diethylenetriamine pentaacetic acid
SWL: shockwave lithotripsy
URS: ureteroscopy

**Keynote message**

Endourological interventions are usually not preferred after the failure of endoureterotomy. However, we obtained successful outcomes after dual stent placement following redo endoureterotomy. This technique of dual ureteral stent placement following laser endoureterotomy was a minimally invasive and effective approach for managing the recalcitrant ureteral stricture after failure of single stent placement following endoureterotomy.

## Introduction

Common causes of ureteral strictures include ischemia, iatrogenic factors, periureteral fibrosis, malignant causes, and congenital factors. In particular, widespread use of URS for treating urolithiasis has led to an increase in the incidence of iatrogenic ureteral strictures. Endoscopic treatments are often used as first‐line interventions; however, they provide poor long‐term success rates. Therefore, reconstructive surgical operations or nephrectomy are preferred in case of strictures longer than 2 cm, ipsilateral renal function of <20%, or failed endoureterotomy.^1^

Ipsilateral dual stents are placed for malignant ureteric obstruction and long and tight strictures to overcome the low success rate of endoscopic treatments in severe cases.^2^, ^3^ In this report, we demonstrate the efficacy of dual stent placement after redo laser endoureterotomy for a persistent ureteral stricture.

## Case presentation

### Clinical history and physical examination

**Fig. 1:**
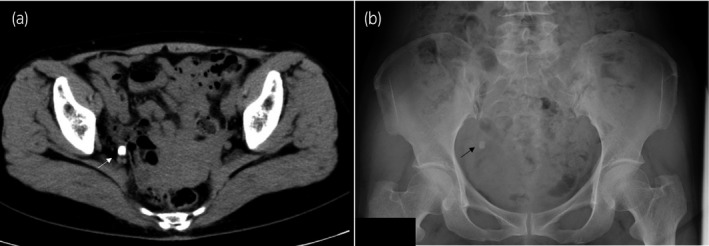
A 7 × 7 mm stone at the right distal ureter detected on plain CT (a; arrowhead) and plain abdominal radiography (b; arrowhead).

A 69‐year‐old woman with a 7 × 7 mm stone in the right distal ureter (Fig. 1) had undergone URS after failed SWL 3 years ago. She had undergone endoureterotomy including ureteral laser incision, balloon dilation (12‐Fr), and single ureteral placement (6‐Fr) for a ureteral stricture after SWL. The removal of the DJ stent, 8 weeks postoperatively, resulted in hydronephrosis and necessitated repeat DJ stent placement. At 32 months after the first operation, the patient was admitted to our clinic.

### Diagnosis

**Fig. 2:**
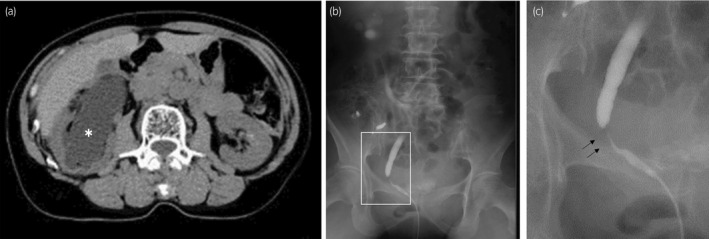
Right renal hydronephrosis and atrophic kidney detected by CT examination (a; asterisk). Retrograde pyelography shows a 15‐mm distal ureteral stricture (b and c; arrowhead).

The patient had severe hydronephrosis, which caused atrophy of her right kidney (Fig. 2a). Severe ureteral stricture was detected in a 15‐mm segment of the right distal ureter by retrograde ureterography (Fig. 2b,c). DTPA renal scintigraphy revealed delayed urine flow from the right kidney, and a split renal function of 27.5%.

### Intervention and follow‐up

**Fig. 3:**
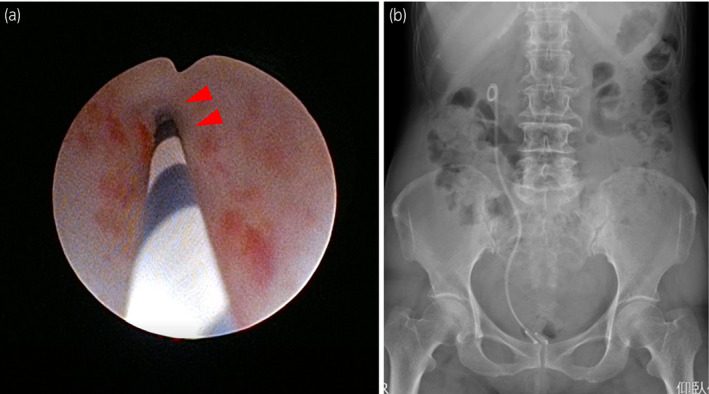
Ureteral stricture seen by URS (a; red arrowhead). Dual stent placement, as visualized by plain abdominal radiograph (b).

**Fig. 4:**
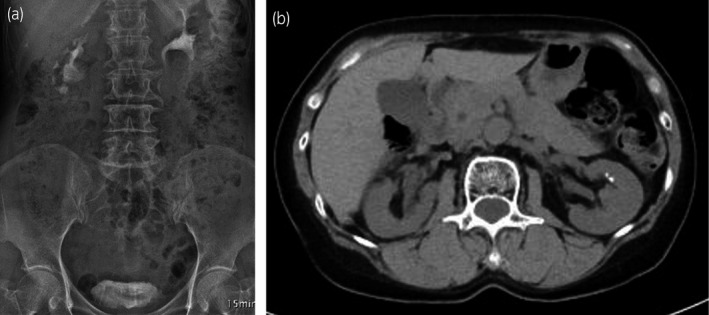
Intravenous urography and CT showing no hydronephrosis at 14 weeks and 34 months after surgery, respectively.

URS using a 6.4‐Fr semi‐rigid ureteroscope (Olympus, Tokyo, Japan) showed a severe ureteric stricture, which barely allowed a single guidewire to pass (Fig. 3a). A redo laser incision was first carefully performed in an anteromedial direction avoiding the iliac vessels, using Holminium YAG Laser (270 μm; Dornier MedTech GmbH, Webling, Germany) at 1.2 Jules X 10 Hertz; the laser pulsed duration was 350 μ/s. The incision extended outward from the ureteral lumen to the periureteral fat, and encompassed 2 mm of normal ureteral tissue proximally and distally. Balloon dilation was subsequently performed to a diameter of 18‐Fr and dual stents of 4.7‐Fr were inserted (Fig. 3b). The surgery was completed in 80 min without complications. Tranilast^®^ (Kissei Pharmaceutical Co., Ltd., Nagano, Japan) was prescribed at a dose of 300 mg per day for 6 months after the operation. Eight weeks later, the dual DJ stents were removed simultaneously; intravenous ureterography performed 14 weeks later showed patency of the ureter without evidence of obstruction (Fig. 4a). However, slight residual hydronephrosis was detected on CT.

## Discussion

The popularity of endoscopic treatments has increased iatrogenic ureteral injuries that lead to strictures. The ureteral damage caused by URS results from various factors, including direct perforation, large caliber ureteral instruments, thermal damage, and lack of operator experience. If inadequately treated, they may result in renal failure.

The most common traditional methods for treating ureteral strictures include segmental resection and ureteral reimplantation; however, with advances in ureteroscopic instruments and techniques, endoureterotomy has become the first choice for managing strictures. The success rates of endoureterotomy for benign ureteral strictures are reportedly 55–85%.^4^ However, endoscopic treatment is likely to fail in cases of strictures longer than 2 cm, ipsilateral renal function <20%, or failed endoureterotomy. Algorithms for managing benign ureteral strictures indicate that endoscopic incision is preferred when stricture lengths are <2 cm and renal function is >20%; open or laparoscopic repair is recommended when stricture lengths are >2 cm or endoureterotomy fails.^1^ Recently, robot‐assisted reconstructive surgery has been reported to be feasible and safe in selected cases. In this case, we performed redo laser endoureterotomy with adequate dilation of up to 18‐Fr, since the first dilation of up to 12‐Fr was probably inadequate.

Ureteric stent placement after endoureterotomy is commonly practiced; however, the optimal stent size and number remain unclear. Certain urologists prefer to insert the largest possible stents; however, large stents may induce ureteral fibrosis, resulting in recurrent strictures owing to mechanical compression. Ipsilateral dual stent placement was originally introduced for malignant ureteric obstruction after single stent placement failure. Recently, it has also become available for patients with rapidly recurring strictures with failed single stent placement after endoureterotomy. In a randomized study, Ibrahim *et al*. demonstrated that dual ureteral stent placement after laser endoureterotomy conferred a higher success rate than single stent placements for ureteral strictures (<1.5 cm). The mechanism is poorly understood; however, dual stents theoretically create a potential space along the ureters between the grooves, and slide relative to one another with peristalsis of the ureter. This relative motion may facilitate durable expansion of the dilated ureteral segment, and prevent ischemia or pressure necrosis of the ureter. In this report, we described the successful treatment of a recurrent ureteral stricture with dual stents after endoureterotomy, following failure of the previous intervention. Further studies are needed to analyze the efficacy of dual stent placement after endoureterotomy for treating recalcitrant strictures.

## Conclusion

Dual ureteral stent placement following endoureterotomy is a minimally invasive and effective approach for the management of recalcitrant ureteral strictures in cases of failed endoureterotomy with a single stent.

## Acknowledgment

The authors would like to thank Editage for the English language review.

## Conflict of interest

The authors declare no conflict of interest.
